# General Anaesthesia Versus Spinal Anaesthesia for Outpatient Total Hip and Knee Arthroplasty: A Randomised Trial Protocol

**DOI:** 10.1111/aas.70283

**Published:** 2026-06-15

**Authors:** Erik Noppa, Karuna Dahlberg, Alex de Leon, Maziar Mohaddes, Anna Philipson, Peter Wildeman, Kristofer F. Nilsson

**Affiliations:** ^1^ School of Medical Sciences, Örebro University Örebro Sweden; ^2^ Department of Anaesthesiology and Intensive Care, Faculty of Medicine and Health Örebro University Örebro Sweden; ^3^ School of Health Sciences, Faculty of Medicine and Health, Örebro University Örebro Sweden; ^4^ Department of Orthopaedics Institute of Clinical Science, The Sahlgrenska Academy, University of Gothenburg Gothenburg Sweden; ^5^ Department of Orthopaedics Hässleholm Hospital Hässleholm Sweden; ^6^ Unit of Orthopedics, Department of Clinical Sciences Malmö, Lund University Malmö Sweden; ^7^ University Health Care Research Center, Faculty of Medicine and Health, Örebro University Örebro Sweden; ^8^ Department of Orthopedic and Hand Surgery Örebro University Hospital Örebro Sweden; ^9^ Department of Cardiothoracic and Vascular Surgery, Faculty of Medicine and Health Örebro University Örebro Sweden

## Abstract

**Introduction:**

Same‐day discharge after primary total hip arthroplasty (THA) and total knee arthroplasty (TKA) is increasing, but the optimal anaesthetic technique for outpatient pathways remains uncertain. Neuraxial anaesthesia is widely recommended based on observational evidence, whereas fast‐track programmes using short‐acting total intravenous anaesthesia may facilitate early mobilisation and discharge. No randomised controlled trial has compared general anaesthesia (GA) with spinal anaesthesia (SA) specifically in outpatient primary THA/TKA using a same‐day discharge target.

**Methods and Analysis:**

GASPS is a pragmatic, multicentre, parallel‐group superiority randomised controlled trial. Adults scheduled for outpatient primary THA or TKA will be randomised 1:1 to GA or SA, with stratification by site and procedure (THA/TKA). The primary outcome is successful same‐day discharge (discharge on the day of surgery) without readmission within 48 h after discharge. The five key secondary outcomes are time to fulfilment of hospital discharge indicators, quality‐of‐recovery (QoR‐15) trajectories over postoperative Days 1–35, patients' qualitative experiences of anaesthesia and recovery, cost per quality‐adjusted life‐year and safety (adverse events); all remaining outcomes—including pain, postoperative nausea and vomiting, opioid consumption, mobilisation and patient‐reported outcomes (EQ‐5D‐5L and HOOS/KOOS)—are exploratory. An embedded qualitative study will explore patient experiences using semi‐structured interviews, and an economic evaluation will compare costs and outcomes from healthcare and societal perspectives. Analyses will follow the intention‐to‐treat principle; a group sequential design with O'Brien‐Fleming boundaries is planned for an interim analysis. Approval has been obtained from the Swedish Medical Products Agency. Written informed consent will be obtained from all participants. Results will be disseminated through peer‐reviewed publications, conference presentations and reporting in the EU Clinical Trials Information System (CTIS).

**Conclusion:**

This study is designed to determine the most effective anaesthetic method for same‐day discharge after primary TKA and THA. With increasing outpatient surgeries, this study will be helpful for optimisation of outpatient processes.

**Trial Registration:**

EU CT/CTIS: 2024‐520127‐89‐00. ClinicalTrials.gov: NCT07334132

**Abbreviations**
AE: adverse event
ASA: American Society of Anesthesiologists
AxMP: auxiliary medicinal product
BMI: body mass index
CTIS: clinical trials information system
EQ‐5D‐5L: european quality of life 5 dimensions 5 level version
EU CT: european union clinical trial
GA: general anaesthesia
GASPS: general anaesthesia versus spinal anaesthesia for success in outpatient primary total hip and knee arthroplasty
GCP: good clinical practice
GDPR: General Data Protection Regulation
HEAP: health economic analysis plan
HOOS: hip disability and osteoarthritis outcome score
ICER: incremental cost‐effectiveness ratio
KOOS: knee injury and osteoarthritis outcome score
NRS: numeric rating scale
NSAID: non‐steroidal anti‐inflammatory drug
OME: oral morphine equivalents
PACU: post‐anaesthesia care unit
POD: postoperative day
PONV: postoperative nausea and vomiting
QALY: quality‐adjusted life year
QoR‐15: quality of recovery‐15
RCT: randomised controlled trial
SA: spinal anaesthesia
SAE: serious adverse event
SAP: Statistical Analysis Plan
SPIRIT: standard protocol items: recommendations for interventional trials
THA: total hip arthroplasty
TIVA: total intravenous anaesthesia
TKA: total knee arthroplasty

## 1 Introduction

### 1.1 Background and Rationale

Primary total hip arthroplasty (THA) and total knee arthroplasty (TKA) are common and effective treatments for osteoarthritis and other degenerative or inflammatory joint diseases, leading to decreased pain, enhanced quality of life and reduced mortality [1, 2, 3]. Outpatient arthroplasty pathways can reduce costs and may reduce healthcare‐associated infection risk, with comparable patient satisfaction and, in selected patients, low complication rates [4, 5, 6]. Same‐day arthroplasty pathways are designed in accordance with Enhanced Recovery After Surgery (ERAS) principles, encompassing preoperative optimisation, opioid‐sparing multimodal analgesia, standardised antiemetic prophylaxis and early mobilisation [7]. However, unsuccessful same‐day discharge and unplanned readmissions remain important safety and implementation challenges [8].

Both spinal anaesthesia (SA) and general anaesthesia (GA) are routinely used for THA/TKA [9]. International consensus statements and observational comparative‐effectiveness research suggest neuraxial techniques may be associated with fewer complications and a higher likelihood of earlier discharge [10, 11, 12]. Conversely, fast‐track pathways using short‐acting total intravenous GA (TIVA) may facilitate earlier mobilisation and shorter length of stay in some contexts [13, 14]. Existing evidence in the outpatient setting is mixed and is largely observational, with potential confounding by patient selection (e.g., higher American Society of Anesthesiologists (ASA) classification among patients receiving GA in routine care) [15, 16].

There is a clear evidence gap regarding the comparative effectiveness of GA versus SA in outpatient primary THA/TKA when the primary goal is successful same‐day discharge and when recovery is assessed using validated patient‐reported recovery instruments.

### 1.2 Objectives

#### 1.2.1 Primary Objective

- Evaluate the effect of SA versus GA on successful same‐day discharge in primary THA/TKA.

#### 1.2.2 Key Secondary Objectives

- Evaluate the effect of SA versus GA on early and intermediate recovery.
- Evaluate the effect of SA versus GA on late and long‐term recovery.
- Describe patient experiences of anaesthesia and postoperative care when undergoing outpatient primary TKA or THA.
- Evaluate the cost‐effectiveness of SA compared to GA from healthcare and societal perspectives.
- Assess the safety of SA and GA.

#### 1.2.3 Exploratory Secondary Objectives

- Evaluate the effect of SA versus GA anaesthesia induction (pre‐recovery) [17].
- Evaluate the effect of SA versus GA on pain and PONV.
- Evaluate the effect of SA versus GA on intraoperative parameters.

An observational cohort of screened patients ineligible for randomisation will be enrolled to allow descriptive analyses and exploratory comparisons with the randomised population.

## 2 Methods and Analysis

### 2.1 Protocol Reporting

This protocol is reported in accordance with the SPIRIT (Standard Protocol Items: Recommendations for Interventional Trials) guidance. A detailed Statistical Analysis Plan (SAP) and Health Economic Analysis Plan (HEAP) will be finalised and made available prior to database lock.

### 2.2 Roles and Responsibilities

The sponsor of the trial is Region Örebro County. The sponsor is responsible for the overall study design, regulatory approvals, trial conduct, data management and reporting.

The principal investigators are responsible for the day‐to‐day conduct of the trial, including participant recruitment, data collection and adherence to the protocol at participating sites.

Trial oversight will be ensured through risk‐based monitoring performed by an independent monitor in accordance with Good Clinical Practice (GCP). No independent data monitoring committee is considered necessary since interventions are standard of care.

### 2.3 Study Design

GASPS is a pragmatic, multicentre, parallel‐group superiority randomised controlled trial with 1:1 allocation to GA or SA. Randomisation will be stratified by site and type of surgery (THA vs. TKA) using permuted blocks. An observational cohort will enrol screened patients who are eligible for outpatient surgery but are not randomised because they decline one of the anaesthetic methods or are deemed unsuitable for either technique; analyses of the observational cohort will be descriptive and exploratory.

### 2.4 Study Setting

Participants will be recruited from specialised orthopaedic surgical units in Sweden with established outpatient THA and/or TKA pathways.

### 2.5 Eligibility Criteria

Inclusion and exclusion criteria are summarised in Table 1.

**TABLE 1:** Key eligibility criteria.

Inclusion criteria	Exclusion criteria
Written informed consent	Indication other than degenerative/inflammatory joint disease (e.g., fracture)
Scheduled for primary THA or TKA for degenerative or inflammatory joint disease	BMI > 35 kg/m^2^
Age 18–80 years at consent	Preoperative opioid use > 20 mg oral morphine equivalents/day
ASA physical status 1–2, or 3 without significant functional impairment	Haemoglobin < 120 g/L
Scheduled surgery start before 13:00 h.	Known bleeding disorder/coagulopathy
Able to communicate in Swedish	Allergy to study‐relevant anaesthetic drugs
	Neurological condition with persistent motor/sensory deficit
	Local infection at spinal injection site
	Determined unsuitable for outpatient surgery and/or trial participation by surgical/anaesthetic team
	Women of childbearing potential not willing to use highly effective contraception or with positive pregnancy test at enrolment
	Unwilling or unable to receive either GA or SA
	Participation in another investigational medicinal product trial within 30 days
	Previous participation in GASPS

### 2.6 Recruitment and Consent

Potentially eligible patients will be identified through routine outpatient arthroplasty scheduling. Written study information will be sent to potentially eligible patients by post ahead of the preoperative anaesthesia assessment, at which the trial will be discussed in person. Participants will provide written informed consent prior to randomisation.

### 2.7 Randomisation and Allocation Concealment

Participants will be randomised 1:1 to GA or SA on the day of surgery via a secure electronic case report form system. Randomisation will use stratified permuted blocks with stratification by site and procedure (THA/TKA) to balance allocation over time. Allocation will be concealed until assignment. Participants will not be informed of allocation until first contact with anaesthesia staff on the day of surgery to reduce expectation effects.

### 2.8 Blinding

Blinding of anaesthesia clinicians and participants is not feasible. Most outcomes are objective time‐based measures (e.g., discharge milestones), recorded in routine care or patient‐reported, minimising but not removing the risk of bias. Data analysis will be performed blinded to treatment assignment until primary analyses are finalised.

### 2.9 Interventions

#### 2.9.1 General Anaesthesia Group

GA will be delivered as TIVA with propofol and remifentanil using target‐controlled infusion or rate‐controlled infusion according to local routine. Airway management will use a supraglottic airway device or endotracheal intubation according to clinical judgement and local practice.

#### 2.9.2 Spinal Anaesthesia Group

SA will be delivered with intrathecal bupivacaine 0.5% (1.0–2.0 mL) and may be combined with intrathecal opioid (up to fentanyl 50 μg or sufentanil 10 μg) according to local routine. Light propofol sedation is permitted at the discretion of the anaesthesiologist. The dose range reflects current Swedish practice and accounts for patient characteristics (height, weight, age) and anticipated surgical duration [18, 19].

#### 2.9.3 Co‐Interventions and Perioperative Care

Perioperative surgical techniques and perioperative care follow established ERAS principles for THA/TKA [7]. The standardised pathway across all sites includes: preoperative patient education, opioid‐sparing multimodal analgesia (paracetamol, NSAID when appropriate, and dexamethasone), standardised PONV prophylaxis, tranexamic acid, antibiotic prophylaxis and protocolised early mobilisation on the day of surgery. Site‐level variation in specific agents or doses is permitted within this framework, consistent with the pragmatic design. Conversions between anaesthetic techniques (e.g., failed spinal requiring GA) will be permitted as clinically necessary and documented.

### 2.10 Outcomes

#### 2.10.1 Primary Outcome

Successful same‐day discharge, defined as discharge from hospital on the day of surgery AND no readmission within 48 h after discharge.

#### 2.10.2 Key and Exploratory Secondary Outcomes

The key secondary outcomes are (1) time from start of surgery to when the patient fulfils indicators of hospital discharge (criteria specified in Table 3), (2) Quality of Recovery (QoR) trajectories, (3) patients' qualitative descriptions of anaesthesia and postoperative recovery, (4) cost per gained quality‐adjusted life‐year (QALY) and (5) safety (adverse events). All other outcomes listed in Table 2 are exploratory, and no multiplicity correction is applied to them. Health economic data will be gathered to capture costs and QALY over a full year to account for seasonal variation, allow short‐term postoperative differences to resolve [20], and support annualised cost‐effectiveness estimates.

**TABLE 2:** Key and exploratory secondary outcomes.

Objectives	Outcomes	Assessment(s)
Key secondary outcomes		
*Evaluate the effect of SA* vs. *GA on early and intermediate recovery*	*Time from start of surgery to when patient fulfils indicators of hospital discharge (criteria specified in* Table 3 *)*	Performed by the discharging orthopaedic surgeon and/or nurse and registered as the time when indicators are fulfilled
*Evaluate the effect of SA* vs. *GA on late postoperative recovery*	*Quality of Recovery (QoR) trajectories*	QoR‐15 at baseline and daily postoperative Day 1–14 and once a week until postoperative day (POD) 35
*Describe patient experiences of anaesthesia and postoperative care*	*Patient's qualitative descriptions of anaesthesia and postoperative recovery*	Semi‐structured interviews with 20–30 participants 14–60 days after surgery
*Evaluate the cost‐effectiveness of SA* vs. *GA*	*Cost per gained quality‐adjusted life‐year (QALY), SA vs. GA*	Quality of life (EQ‐5D‐5L, HOOS/KOOS): questionnaires at baseline, 4 weeks, 6 and 12 months Intervention costs: time, material and personnel across stages of anaesthesia Healthcare consumption (inpatient, outpatient, primary care): administrative data, first year Prescribed medication: administrative data and medical records, first year Productivity and informal care: patient questionnaires at baseline, 4 weeks, 6 and 12 months
*Assess the safety of SA and GA*	*Adverse events (AE) and Serious AE (SAE)*	Reported by study staff (see Safety section), from start of anaesthesia to 3 days post‐surgery
Exploratory secondary outcomes		
Preoperative care and anaesthesia		
Evaluate the effect of SA vs. GA on anaesthesia induction (early recovery)	Time from start of anaesthesia to start of surgery	Times recorded by the anaesthesia or study staff
Postoperative recovery		
Evaluate the effect of SA vs. GA on early and intermediate recovery	Time from start of surgery to discharge from PACU (criteria specified in Table 3)	Assessed by the postoperative recovery unit nurse and recorded as the time of discharge
	Time from the start of surgery to hospital discharge	Assessed by the discharging orthopaedic surgeon and/or nurse and recorded as the time of discharge
	Time from start of surgery to first successful mobilisation by walking with tolerable pain	Assessed by nurse or physiotherapist; successful if the patient can walk with tolerable pain
Pain and PONV		
Evaluate the effect of SA vs. GA on pain and PONV during early, intermediate, late and long‐term recovery	Rated pain	NRS for pain at rest and mobilisation during early and intermediate recovery and as items 11 and 12 of QoR‐15, POD 1–35
	Use of analgesics (opioids converted to oral morphine equivalents, OME)	Registered by nurse for early and intermediate recovery and reported by the patient after discharge
	Postoperative nausea and vomiting (PONV)	Registered by nurse during early and intermediate recovery and as an item in QoR‐15, POD 1–35
	Patient‐reported outcomes of surgical intervention	HOOS or KOOS at 4 weeks, 6 and 12 months postoperatively
Safety (additional)		
	Incidence of predefined adverse outcomes (specified in the full trial protocol)	Collected from start of anaesthesia to end of follow‐up 2 by patient or study staff
	Need of blood transfusion	Blood transfusion recorded in medical records
Surgical outcomes		
Evaluate the effect of SA vs. GA on intraoperative parameters	Duration of surgery	Intraoperatively recorded data on start and end of surgery
	Intraoperative blood loss	Data from anaesthesia records

*Note:* Outcomes shown in italics are the five key secondary outcomes; all remaining outcomes are exploratory.

Abbreviations: AE, adverse event; AxMP, auxiliary medicinal product; EQ‐5D‐5L, European Quality of Life 5 Dimensions 5 Level version; GA, general anaesthesia; HOOS, Hip disability and Osteoarthritis Outcome Score; KOOS, Knee injury and Osteoarthritis Outcome Score; NRS, Numeric Rating Scale; OME, oral morphine equivalents; PACU, post‐anaesthesia care unit; POD, postoperative day; PONV, postoperative nausea and vomiting; QALY, quality‐adjusted life year; QoR‐15, Quality of Recovery‐15; SA, spinal anaesthesia; SAE, serious adverse event; THA, total hip arthroplasty; TKA, total knee arthroplasty.

**TABLE 3:** PACU discharge criteria and indicators of possible discharge from hospital.

**PACU discharge criteria**	
Respiration	SpO2 ≥ 93% with < 4 L O2
Circulation	BP 80%–120% of preoperative values HR 45–100/min
Temperature	≥ 36°C, 0°C
Activity	Ability to move feet if SA
Pain	NRS ≤ 4
**Indicators of possible discharge from hospital**	
Pain	NRS ≤ 4 at rest
Ambulation	Able to walk with tolerable pain Able to walk one flight of stairs (if present at home)
Surgical dressings	No signs of active bleeding
Labs	Hb ≥ 100 g/L
Voiding	Normal
Radiological examination of prosthesis	Performed

Key and exploratory secondary outcomes are listed in Table 2.

### 2.11 Schedule of Assessments

See Table 4.

**TABLE 4:** Summary of the main data collection time points and instruments.

Measurements and activities	Timepoint
Assessment of inclusion and exclusion criteria	Screening
Informed consent	
Patient characteristics	
Medical history	
Opioid use	
Type of surgery (planned)	
Pregnancy test (if applicable)	
Assessment of inclusion and exclusion criteria	
EQ‐5D‐5L, HOOS/KOOS	Baseline
QoR‐15	
Productivity	
Randomisation	Preoperative care and anaesthesia
Administered drugs (AxMP, concomitant medications)	
Needle used, spinal medications and dosage (SA group)	
TIVA protocol used	
Time for start of anaesthesia	
Time of initiation and completion of sterile draping and skin disinfection	
Staff present during anaesthesia induction	
Predefined adverse outcomes	
AEs/SAEs	
Time for start of surgery	Intraoperative (surgery) Day 0
Surgical method	
Bleeding	
Dose of Propofol and remifentanil	
Dose of systemic and local tranexamic acid	
Administered drugs (AxMP, concomitant medications)	
Airway management	
Anaesthesia staff present during surgery	
Time for end of surgery	
Predefined adverse outcomes	
AEs/SAEs	
*Follow‐up 1*	
Pain levels (NRS)	Immediately postoperative (early recovery)
Administered drugs (AxMP, concomitant medications)	
Predefined adverse outcomes	
AEs/SAEs	
Pain levels (NRS) at 1 h after surgery and highest during the first postoperative hour	First postoperative hour (early recovery)
Administered drugs (AxMP, concomitant medications)	
PONV	
Dizziness	
Predefined adverse outcomes	
AEs/SAEs	
PACU discharge criteria (Criteria specified in full in the trial protocol)	Discharge from PACU to the ward
Pain levels (NRS) at discharge and highest during this phase	
Time of discharge	
Administered drugs (AxMP, concomitant medications)	
Analgesics use	
Predefined adverse outcomes	
AEs/SAEs	
The time of first mobilisation	Intermediate recovery (Ward)
Transfusions (number of units transfused)	
Deviations from routine care at site, as described in protocol	
The time when patient fulfils indicators for end of phase III, intermediate recovery (Criteria specified in full in the trial protocol)	
Administered drugs (AxMP, concomitant medications)	
Analgesics use	
Predefined adverse outcomes	
AEs/SAEs	
Time of discharge from hospital.	At discharge from hospital
Predefined adverse outcomes	
AEs/SAEs	
*Follow‐up 2—Conducted both in discharged and non‐discharged patients*	
QoR‐15, Analgesics use	POD 1–14 (daily)
Patient reported predefined adverse outcomes (Specified in full in the trial protocol)	
AEs/SAEs up to POD 3	
Predefined adverse outcomes	
Readmission monitoring	
QoR‐15, Analgesics use	POD 15–35 (weekly ±2 days)
Patient reported predefined adverse outcomes (Specified in full in the trial protocol)	
Semi‐structured interviews (14–60 days postoperatively)	
AEs/SAEs	
Predefined adverse outcomes	
Readmission monitoring	
EQ‐5D‐5L, HOOS/KOOS, Productivity questionnaire	4 weeks postoperative (±7 days)
Healthcare consumption data	
AEs/SAEs	
Predefined adverse outcomes	
Readmission monitoring	
*Follow‐up 3*	
EQ‐5D‐5L, HOOS/KOOS, Productivity questionnaire	6 months postoperative (±14 days)
Prescription and healthcare consumption data	
*Follow‐up 4*	
EQ‐5D‐5L, HOOS/KOOS, Productivity questionnaire	12 months postoperative (±14 days)
Prescription and healthcare consumption data	

Abbreviations: AE, adverse event; AxMP, auxiliary medicinal product; EQ‐5D‐5L, European Quality of Life 5 Dimensions 5 Level version; HOOS, Hip disability and Osteoarthritis Outcome Score; KOOS, Knee injury and Osteoarthritis Outcome Score; NRS, Numeric Rating Scale; PACU, post‐anaesthesia care unit; POD, postoperative day; PONV, postoperative nausea and vomiting; QoR‐15, Quality of Recovery‐15; SA, spinal anaesthesia; SAE, serious adverse event; TIVA, total intravenous anaesthesia.

### 2.12 Sample Size

The trial will enrol 600 participants (300 per group). This sample size provides 80% power at a two‐sided 5% significance level to detect an absolute difference of 10 percentage points in successful same‐day discharge between groups, with expected success rates of 70% (GA) and 80% (SA). To account for potential loss to follow‐up, the sample size allows for approximately 1% attrition (*n* = 6 participants). The effect size was informed by prior literature, retrospective cohort data and clinical consensus [21].

### 2.13 Data Collection and Management

Trial data will be captured in an electronic case report form. Data quality will be supported by built‐in checks, monitoring and source data verification according to risk‐based monitoring principles. Data will be pseudonymised using a trial‐specific code; the re‐identification key will be stored locally at each site. All data handling will comply with the General Data Protection Regulation (GDPR) and applicable Swedish data protection legislation.

### 2.14 Statistical Analysis

Analyses will follow the intention‐to‐treat principle. The primary analysis of the primary outcome will compare the proportion of successful same‐day discharge between groups using a chi‐square test (or Fisher's exact test if appropriate). A prespecified adjusted analysis using logistic regression including treatment group, type of surgery and study site as fixed effects will also be performed, consistent with the stratified randomisation; both analyses will be reported. Effect estimates will be reported as risk difference and odds ratio with 95% confidence intervals. Analyses adjusted for other prespecified baseline variables will be performed as appropriate. The full analytical approach is prespecified in the statistical analysis plan, which will be finalised prior to database lock.

Continuous secondary outcomes will be analysed using independent *t*‐tests or non‐parametric alternatives as appropriate, and regression models for adjusted analyses. Repeated‐measures outcomes (e.g., QoR‐15 trajectories) will be analysed using mixed‐effects models including treatment group, time and group‐by‐time interaction, with participant‐level random effects and, where appropriate, site‐level effects. Binary safety endpoints will be analysed using chi‐square/Fisher's exact tests and logistic regression. Time‐to‐event endpoints may be analysed using Kaplan–Meier methods and Cox models where appropriate. A two‐sided significance level of 0.05 will be employed.

An interim analysis of the primary outcome is planned after approximately 300 participants have completed primary endpoint assessment to allow early stopping for both futility and safety. A group sequential design with O'Brien‐Fleming boundaries will be used to preserve the overall two‐sided type I error at 0.05. Exact stopping boundaries will be based on the observed information fraction at the interim analysis.

### 2.15 Embedded Qualitative Study

A purposive sample of approximately 20–30 trial participants (aiming for at least 10 from each group) will be invited to take part in semi‐structured interviews 14–60 days after surgery. Interviews will explore experiences of anaesthesia, outpatient discharge and postoperative recovery. Interviews will be audio‐recorded, transcribed verbatim and analysed using reflexive thematic analysis with iterative coding and theme development [22, 23].

### 2.16 Health Economic Evaluation

A cost‐utility analysis will be performed with effects measured as quality‐adjusted life years (QALYs) derived from EQ‐5D‐5L using Swedish preference weights. Incremental cost‐effectiveness ratios will compare GA versus SA. Costs will include intervention‐related resource use, healthcare utilisation, prescribed medications and productivity losses. Analyses will be conducted from both healthcare and societal perspectives. Deterministic and probabilistic sensitivity analyses will explore uncertainty. Secondary cost‐effectiveness analyses will use successful same‐day discharge and HOOS/KOOS as effect measures.

### 2.17 Patient and Public Involvement

Patients and/or the public were not involved in the design of this protocol. Plans for patient‐facing dissemination include a lay summary reported in CTIS and accessible summaries of findings after trial completion.

## 3 Ethics and Dissemination

### 3.1 Ethics Approval and Consent

The study has received regulatory approval from the Swedish Medical Products Agency (EU CT number: 2024‐520127‐89‐00) following a favourable opinion from the Swedish Ethical Review Authority. The trial complies with Regulation (EU) No. 536/2014.

Informed consent will be obtained by a physician at each participating site. Potential participants will receive both oral and written information about the study during the preoperative anaesthesia assessment or at a dedicated study visit prior to surgery.

Participants will be given time to consider participation and to ask questions before providing written informed consent. Consent will be obtained prior to any study‐specific procedures, including randomisation. Consent includes permission to collect and use study data from medical records and patient‐reported outcomes. No biological samples will be collected for research purposes.

Participants will be informed that participation is voluntary and that they may withdraw from the study at any time without any consequences for their clinical care. Participants may also be asked to provide additional consent for participation in the embedded qualitative study involving semi‐structured interviews.

### 3.2 Safety Considerations

Both GA and SA are established standard‐of‐care anaesthetic techniques for THA/TKA. Eligibility criteria select patients suitable for outpatient pathways to minimise risk. Adverse events (AEs) and serious adverse events (SAEs) will be defined and assessed according to standard clinical trial definitions and recorded systematically from anaesthesia start to postoperative Day 3. Predefined adverse outcomes will be captured during follow‐up windows defined in the trial protocol.

### 3.3 Confidentiality

Personal data will be processed in accordance with GDPR and Swedish data protection laws. Published results will be reported in aggregate, and no individual participant will be identifiable.

### 3.4 Dissemination

Findings will be disseminated through peer‐reviewed journals, presentations at national and international meetings and reporting of summary results in CTIS, including a lay summary. Secondary publications are planned for recovery trajectories, qualitative findings, health economic results and longer‐term outcomes.

### 3.5 Data Sharing

Individual participant data will not be publicly available due to privacy and regulatory restrictions. De‐identified data may be made available upon reasonable request to the corresponding author, subject to approval by the sponsor and relevant authorities, and in accordance with applicable data protection regulations.

The SAP and HEAP will be finalised and made publicly available in an open‐access repository prior to database lock and analysis. The statistical code used for the primary analyses will be made available following publication of the results.

## 4 Trial Status

At the time of submission, the trial status is: not yet recruiting.

Two sites, Lindesberg Hospital and Hässleholm Hospital, are participating in the study from the start. Additional sites may be added. Protocol versions are as stated on the title page. Recruitment start and end dates will be reported in upcoming reports.

## 5 Discussion

The choice of anaesthetic methods in TKA and THA has changed little over the last decades despite reduced hospitalisation. Observational studies and register‐based analyses have suggested advantages for neuraxial anaesthesia in terms of perioperative complications and length of stay [12, 24], yet recent cohort data from ambulatory settings show comparable outcomes regardless of technique [9, 25], and two small randomised trials have even indicated faster recovery with TIVA [13, 14].

GASPS is designed as a pragmatic trial; this means that the study is conducted as close to routine clinical care as possible at centres with established outpatient arthroplasty programmes. This design philosophy has several advantages as it enhances external validity, facilitates recruitment, and ensures that the findings are applicable to implementation. Stratification by site and procedure (THA/TKA) in the randomisation further reduces the risk that site‐level variation in co‐interventions confounds the primary comparison, and sensitivity analyses adjusting for important co‐interventions will be pre‐specified in the SAP.

However, some standardisations are required to maintain features that define modern GA and SA in this context while allowing for variation in elements that do not alter the nature of the techniques [26].

Inclusion and exclusion criteria for this study focus on including all patients that may participate in the study but exclude for safety, unsuitability for either SA or GA, or factors that make successful same day discharge unlikely. Unicompartmental knee arthroplasty is not included in the trial, as it differs from TKA in surgical invasiveness and recovery profile. Preoperative opioid use is one such factor that has been described to increase the risk of unsuccessful same day discharge in the literature, and most Swedish outpatient pathways exclude all patients with higher doses of preoperative opioids [21].

### 5.1 Choice of Primary Endpoint

We chose successful same‐day discharge as the primary outcome because it reflects the goal of outpatient arthroplasty pathways and is meaningful to patients, clinicians and healthcare systems. Unlike surrogate measures such as time to first mobilisation or pain scores at a single time point, same‐day discharge integrates multiple domains of recovery—pain control, haemodynamic and respiratory stability, motor function, absence of surgical complications and patient willingness—into a single, pragmatic endpoint. A composite definition requiring no readmission within 48 h was added to guard against premature discharge that merely shifts morbidity to a later time point [4, 16, 27]. The limitations of this endpoint include reasons for not being discharged that are not entitled to medical and patient‐related causes and that there is some subjectivity included in the discharge decision.

### 5.2 Quality of Recovery‐15 as a Key Secondary Outcome

QoR‐15 was selected as the principal patient‐reported recovery outcome because it captures a multidimensional construct—physical comfort, emotional state, pain, physical independence and patient support—that is not adequately reflected by single‐item measures such as pain scores alone [28]. QoR‐15 has been validated in multiple languages and surgical populations including outpatient orthopaedic surgery, and has an established minimal clinically important difference [29, 30, 31, 32].

In GASPS, QoR‐15 is administered daily from postoperative Day 1–14 and weekly until Day 35. This extended assessment is a strength of the trial, as most studies have measured QoR‐15 only at 24 h or at hospital discharge [31], similar findings were also identified when reviewing assessment of recovery in research [33]. In both studies, the researchers suggest that recovery should be measured several times during recovery [31, 33]. Patients are willing to report daily on how they experience their recovery, and digital assessment has a higher response rate compared to paper‐based assessment [34, 35]. The repeated‐measures design will enable characterisation of the recovery trajectory and identification of the time point at which any potential between‐group difference appears or resolves. This trajectory‐based analysis addresses the need to move beyond single‐snapshot recovery assessments and to examine quality of care beyond traditional markers of morbidity and/or mortality [17, 36]. To mitigate this, the digital follow‐up platform sends reminders to support frequent QoR‐15 completion. Repeated‐measures outcomes will be analysed using mixed models, which provide valid estimates under the assumption that data are Missing At Random (MAR). Should the proportion of missing data exceed a threshold specified in the SAP, sensitivity analysis using multiple imputation will be performed.

### 5.3 Assumed Treatment Effect and Sample Size

The sample size is based on an absolute difference of 10 percentage points in successful same‐day discharge (70% vs. 80%). This estimate was informed by published discharge rates from outpatient arthroplasty cohorts and retrospective comparisons of anaesthetic technique, but same‐day discharge rates vary widely [4, 9, 16, 21].

A 10‐percentage‐point difference was both in line with earlier studies and deemed clinically meaningful in consultation between orthopaedic surgeons and anaesthesiologists at participating centres. Smaller differences, whereas potentially statistically detectable with a larger sample, are unlikely to change clinical practice on their own.

### 5.4 Blinding

Full blinding of participants is not feasible given the nature of the interventions, and this represents an acknowledged limitation of the trial. Since the majority of outcomes are either recorded as part of routine care or are patient‐reported, the associated risk of bias is considered small. Blinding of statistical analyses will be maintained as a mitigation strategy. We consider transparent acknowledgement of this limitation preferable to artificially constraining patient–provider interactions.

### 5.5 Qualitative Study

The embedded qualitative study is designed to contextualise the quantitative findings by exploring patients' experiences of anaesthesia, discharge process and early recovery. Although the quantitative methods can establish whether the interventions work, qualitative methods are needed to add deeper understanding of how and why outcomes differ, to capture aspects of the patient experience that questionnaires may overlook, and to provide ideas for further studies [37].

Previous qualitative studies in the fast‐track arthroplasty context have shown that patients' experiences of early discharge are shaped by factors such as preparedness, sense of safety, pain management and social support—themes that are difficult to capture with purely quantitative instruments [34, 38, 39]. The purposive sampling strategy targeting 20–30 participants (at least 10 per group) is guided by the principle of information power [40]. Reflexive thematic analysis was chosen for its flexibility and theoretical accessibility [22, 23]. One limitation will be the inherited risk of selection of patients willing to participate in this part of the study.

## 6 Conclusions

This study is designed to determine the most effective anaesthetic method for same‐day discharge after primary THA and TKA including embedded health economic and qualitative analyses of patient experiences. In the era of increasing outpatient surgeries, this study will be helpful for anaesthesia providers and outpatient centres for optimisation of their processes.

## Author Contributions

**Erik Noppa:** conceptualisation, methodology, writing – original draft, writing – review and editing, project administration. **Karuna Dahlberg:** conceptualisation, methodology, writing – review and editing. **Alex de Leon:** conceptualisation, methodology, writing – review and editing. **Maziar Mohaddes:** methodology, writing – review and editing. **Anna Philipson:** methodology, writing – review and editing. **Peter Wildeman:** conceptualisation, writing – review and editing. **Kristofer F. Nilsson:** conceptualisation, methodology, supervision, funding acquisition, writing – review and editing. All authors read and approved the final version of the manuscript.

## Funding

This work was supported by ALF funding Region Örebro County grant number OLL‐1010516 and OLL‐993657.

## Conflicts of Interest

The authors declare no conflicts of interest.

## Data Availability

Data sharing not applicable to this article as no datasets were generated or analysed during the current study.
